# Cardiac Glycosides as Autophagy Modulators

**DOI:** 10.3390/cells10123341

**Published:** 2021-11-28

**Authors:** Jan Škubník, Vladimíra Svobodová Pavlíčková, Jana Psotová, Silvie Rimpelová

**Affiliations:** Department of Biochemistry and Microbiology, University of Chemistry and Technology, Technická 3, 166 28 Prague, Czech Republic; jan.skubnik@vscht.cz (J.Š.); vladimira.pavlickova@vscht.cz (V.S.P.); jana.psotova@vscht.cz (J.P.)

**Keywords:** bufalin, digoxin, ouabain, peruvoside, Na^+^/K^+^-ATPase, autosis, LC3-II, Beclin 1, mTOR

## Abstract

Drug repositioning is one of the leading strategies in modern therapeutic research. Instead of searching for completely novel substances and demanding studies of their biological effects, much attention has been paid to the evaluation of commonly used drugs, which could be utilized for more distinct indications than they have been approved for. Since treatment approaches for cancer, one of the most extensively studied diseases, have still been very limited, great effort has been made to find or repurpose novel anticancer therapeutics. One of these are cardiac glycosides, substances commonly used to treat congestive heart failure or various arrhythmias. Recently, the antitumor properties of cardiac glycosides have been discovered and, therefore, these compounds are being considered for anticancer therapy. Their mechanism of antitumor action seems to be rather complex and not fully uncovered yet, however, autophagy has been confirmed to play a key role in this process. In this review article, we report on the up-to-date knowledge of the anticancer activity of cardiac glycosides with special attention paid to autophagy induction, the molecular mechanisms of this process, and the potential employment of this phenomenon in clinical practice.

## 1. Introduction

According to the estimated projection of US cancer incidence and death in 2040 published by Rahib et al. [1] in 2021, the incidence of cancer will generally increase. In comparison with 2020, the number of diagnosed cancer cases in 2040 will increase by nearly 150,000 to 1,881,000. Although the incidence will increase, the number of deaths will most probably be lower than in 2020. The estimate of 410,000 deaths is, however, worrying. The ratio of deaths makes cancer still one of the most threatening diseases. Currently, not a few cancer-related deaths in 2021 will be indirectly connected with reduced access to health care due to the coronavirus disease 2019 (COVID-19) pandemic [2]. The COVID-19 pandemic has also shown that besides developing new drugs and treatment strategies, it is also possible to search for useful active compounds among already approved drugs, which are used to treat other indications [3]. This approach, called “drug repositioning”, has also long been implemented in cancer treatment. It enables a partial speeding up of the process of a drug’s clinical evaluation since basic information on the therapeutic parameters of a repurposed drug is already known [4]. As an example, cardiac glycosides (CGs) have emerged as potentially very useful anticancer therapeutics.

CGs are widely distributed substances of both plant and animal origin that have found an application in the treatment of cardiovascular diseases, especially heart failure. Their effects have been known since ancient times [5]. Not only were they used for healing, but also as a part of arrow poisons [6,7]. First, CGs have been incorporated into modern medicine by an English doctor and botanist William Withering, who summarized his findings in his book “An account of the foxglove, and some of its medical uses” published in 1785 [8]. Almost 200 years later, in 1967, Shiratori [9] reported on the inhibition of neoplastic cells by CGs for the first time. Since then, the interest in CGs as potential anticancer chemotherapeutics has increased.

Chemically, CGs are steroid compounds, and their basic structure consists of three major parts: a steroid core, an unsaturated lactone ring, and a carbohydrate moiety. The steroid core is the pharmacophore common to all CGs and consists of four rings: A, B, C, and D. The A/B and C/D rings are in *cis*-conformation, which differs from glucocorticoids, mineralocorticoids, and sex hormones, and the B/C rings occur in *trans*-conformation [10]. On the A ring in position C-3, there is a carbohydrate moiety, which is mostly d-glucose, d-digitoxose, d-mannose, or d-galactose [11]. Based on the nature of the lactone on the D ring in position C-17, CGs can be classified into cardenolides and bufadienolides. The first group, cardenolides, the examples of which are digoxin, digitoxigenin, or ouabain have an unsaturated five-membered 2-furanone ring in position C-17 and predominantly occur in plants [12]. In contrast, bufadienolides, such as bufalin or cinobufagin, are characterized by an unsaturated six-membered 2-pyrone ring and are mainly found in animals, especially in amphibians [13].

## 2. Cardiac Glycosides and Sodium-Potassium ATPase

CGs derive their biological activity from binding to sodium-potassium ATPase (NKA), a heteromeric protein complex located on the cytoplasmic membrane of eukaryotic cells, which maintains Na^+^ and K^+^ homeostasis in a cell. In 1957, the existence of NKA was first reported in the carb nerve [14]. In terms of enzyme activity, it was originally classified as hydrolase [EC 3.6.3.9], however, according to the new classification, it belongs to translocases [EC 7.2.2.13]. Similar to other enzymes from this class, NKA uses energy from adenosine triphosphate (ATP) hydrolysis to transport Na^+^ and K^+^ ions across the cytoplasmic membrane against their concentration gradients. During each cycle, NKA binds three Na^+^ ions from the intracellular space and exports them outside of a cell. Then, it binds two K^+^ ions from the extracellular space and transports them inside the cell. Thus, NKA is involved in maintaining ionic and osmotic balance in cells. Importantly, during the ion exchange, the NKA conformation transits between two states E1 and E2 which have different affinities for Na^+^, K^+^, and ATP [15].

Structurally, NKA usually forms a dimer composed of two non-covalently linked α and β subunits. The α subunit consists of ten transmembrane helixes (M1–M10) and it is the catalytic subunit of NKA [16]. The β subunit is a glycoprotein with a regulatory function; it enables the transport of the α-subunit to the cytoplasmic membrane, in which it stabilizes the complex [17,18]. Both subunits occur in multiple isoforms, the expression of which differs depending on the type of tissue. Specifically, the α subunit occurs in four different isoforms (α1–α4) and the β subunit exists in three isoforms (β1–β3). The most common combination of the isoforms in humans is α1β1 [19]. In addition to α and β subunits, in some cases, a protein from the FXYD family is found in the NKA complex [20]. An FXYD subunit may be present in seven different isoforms (FXYD1–7) all of which share a common conservative amino acid sequence of l-Phe, X, l-Tyr, l-Asp (X stands for l-Glu, l-His, l-Phe, l-Thr, or l-Tyr). FXYD subunits function as modulators of the ion transport function of NKA [19,20,21].

Ion transport by NKA is a process, which is targeted by CGs in terms of their cardiotonic action. During NKA inhibition, CGs decrease the intracellular concentration of K^+^ ions and increase the intracellular concentration of Na^+^ ions. To maintain ion homeostasis after this inhibition, the Na^+^/Ca^2+^-exchanger activity is suppressed, which leads to an augmented concentration of intracellular Ca^2+^ and enhanced muscle contractility. This series of actions is called the positive inotropic effect of CGs [22,23]. While the cardiotonic activity of CGs is based only on the inhibition of NKA pumping activity, the antineoplastic CG activity is a result of a complex concentration-dependent mechanism of action. Higher CG concentrations (hundreds of nM) cause inhibition of NKA and subsequently induce apoptosis [24,25]. This is partially connected with the inhibition of NKA pumping activity. It has been found that the physiological intracellular concentration of K^+^ ions suppresses apoptosis. As the transport of K^+^ ions inside cells is abolished during NKA inhibition, caspases are activated after K^+^ depletion, followed by a release of cytochrome c, leading to activation of DNA-cleaving nuclear endonucleases [26]. Besides K^+^ ions, Ca^2+^ ion homeostasis modulation induced by NKA inhibition can also lead to apoptosis. In particular, increased levels of intracellular Ca^2+^ may lead to the activation of a cysteine protease calpain, which has been described as being involved in cell death linked with various neurodegenerative conditions [27,28]. In addition to modulation of K^+^ and Ca^2+^ levels by CGs, increased extracellular Na^+^ levels are also a potent apoptosis inducer since they reduce the activity of the Na^+^/H^+^-exchanger (NHE). NHE activity is increased in tumor cells and its suppression causes intracellular acidification leading to apoptosis [29,30].

**Figure 1:**
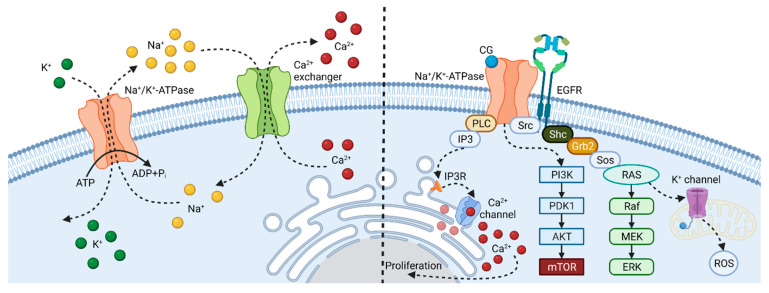
Two main physiological functions of Na^+^/K^+^-ATPase (NKA). NKA ensures the transport of two K^+^ ions inside and three Na^+^ ions outside the cell. Thereby, it simultaneously impacts the homeostasis of Ca^2+^ ions, because the Ca^2+^ exchanger needs the right level of Na^+^ ions for the antiport of Ca^2+^ ions from the cell. NKA is also a key receptor of a large signalosome. If NKA is activated by a ligand (e.g., a CG), non-receptor tyrosine kinase (Src) is activated to phosphorylate the epidermal growth factor receptor (EGFR). Through adapter proteins, Src homology 2 domain-containing transforming protein (Shc)/growth factor receptor-bound protein 2 (Grb2)/son of sevenless protein (Sos), the rat sarcoma protein (Ras) pathway is triggered, which means a sequential activation of serine/threonine kinase (Raf), mitogen-activated protein kinase (MEK) and extracellular signal-regulated kinase (ERK) pathway. Ras also potentiates mitochondrial reactive oxygen species (ROS) production via the opening of the mitochondrial K^+^ channel. Besides Src/EGFR/Ras signalosome part, NKA interacts also with phospholipase C (PLC), which, when activated, hydrolyzes phosphatidylinositol-4,5-bisphosphate to inositol-1,4,5-triphosphate (IP3), which interacts with the IP3 receptors in the endoplasmic reticulum (ER). Interaction with this receptor opens the Ca^2+^ channel on ER, releasing Ca^2+^ ions into the cytosol. Disruption of Ca^2+^ ion homeostasis can lead to cell proliferation, differentiation, or apoptosis. In addition, phosphatidylinositol-3-kinase (PI3K) is also linked to NKA in the signalosome. Its activation leads to protein kinase B (Akt)/mammalian target of rapamycin (mTOR) pathway trigger and, thereby, to autophagy and apoptosis modulation. Created with BioRender.com (accessed on 22 October 2021).

The mechanisms of cell death induction and, thus, also the anticancer mechanisms of CG action, are, however, only partially based on causing ion imbalance. There is a much more important reason for such antiproliferative activity of CGs. At low concentrations (units and tens of nM), CGs affect the non-pumping function of NKA (Figure 1), namely its receptor activity as a part of a signalosome complex [31,32]. NKA signalosome is a multiple protein signaling complex located in cholesterol- and caveolin-1-formed caveolae of the plasma membrane. The core of the signalosome is created by three α and two β subunits of NKA [32]. NKA is linked to other receptors, such as the epidermal growth factor receptor (EGFR), and intracellular signaling pathway transducers, such as proto-oncogene tyrosine-protein kinase Src (Src), rat sarcoma virus protein (Ras), phospholipase C (PLC), and phosphoinositide 3-kinase (PI3K). All these receptors and signal transducers form the NKA signalosome, the effects of which are potently modulated by CGs. The interaction of CGs with NKA in the signalosome causes a conformational change of the signalosome and, thus, Src activation. These tyrosine kinases can link receptors lacking intrinsic kinase activity to receptor tyrosine kinases such as proximal EGFR. Through adaptor proteins, the signal is transmitted to the Ras-Raf-MEK-MAPK (Ras/rapidly accelerated fibrosarcoma protein/mitogen-activated protein kinase kinase/mitogen-activated protein kinase) signaling cascade and, thus, transcription factors that regulate gene expression are activated [33,34,35,36]. Activated Ras protein can also induce the mitochondrial production of reactive oxygen species (ROS) caused by the opening of ATP-sensitive potassium channels in mitochondria [37,38]. In parallel, PLC hydrolyzes phosphatidylinositol-4,5-bisphosphate to inositol 1,4,5-trisphosphate (IP_3_) which interacts with the IP_3_ receptors on the endoplasmic reticulum (ER). Interaction with this receptor opens the Ca^2+^ channel in ER, releasing Ca^2+^ ions into the cytosol. Disruption of Ca^2+^ ion homeostasis can lead to cell proliferation, differentiation, or apoptosis [39,40,41]. In addition, the PI3K-Akt-mTOR (PI3K/protein kinase B/mammalian target of rapamycin) signaling pathway is also connected to NKA. PI3K is activated by increased expression of the NKA α-subunit and phosphorylates Akt, which activates mTOR. This signaling pathway is an important component in the processes of apoptosis and autophagy [42,43,44,45].

## 3. Autophagy

**Figure 2:**
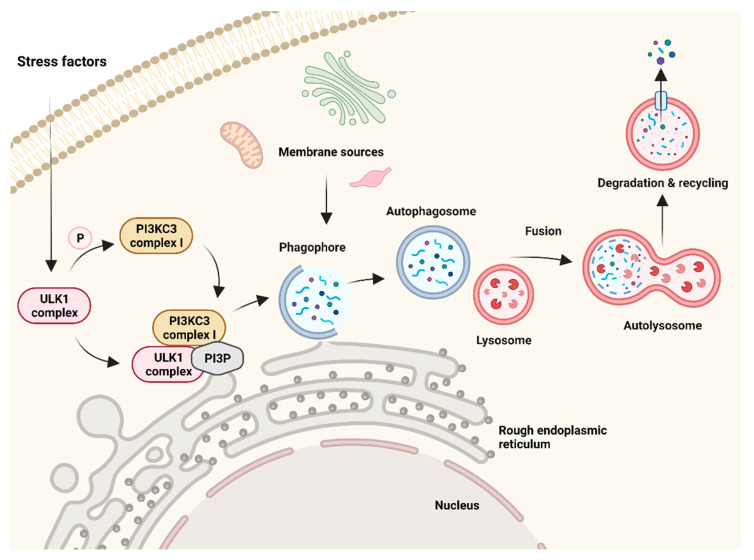
The process of autophagy. Exposure to stress leads to activation of Unc-51 like autophagy activating kinase 1 (ULK1) complex, phosphorylation of phosphatidylinositol 3-kinase catalytic subunit type 3 (PI3KC3), and formation of ULK1, PI3KC3, phosphatidylinositol 3 phosphate (PI3P) complex in the membrane of the endoplasmic reticulum (ER). Next, a phagophore is formed from various membrane sources, including the ER membrane. The phagophore maturates into an autophagosome (a vesicle containing cargo to be degraded), which fuses with a lysosome to create an autolysosome. In this vesicle, the cargo is degraded by lysosomal lytic enzymes and the lysis products can be recycled. Created with BioRender.com (accessed on 4 November 2021).

Autophagy is an adaptive catabolic process, which occurs in cells both at physiological conditions and after exposure to some kind of stress. Physiologically, it serves as a damage repair and cellular recycling process promoting cell survival, but under certain conditions, it might also lead to cell death. Such conditions are mainly lasting exposure to stress factors, such as chemotherapy or radiation, starvation, hypoxia, or growth factor deprivation. The process of autophagy (Figure 2) begins with the formation of a phagophore, which is an initiating complex of crucial autophagy-related (ATG) proteins, and a small, flattened, cup-shaped membrane structure called isolation membrane. The very first step of the autophagy initiation is the recruitment of the ATG proteins to a specific site within a cell, which is called the phagophore assembly site (PAS). This site is in close contact with ER exit sites, which are specialized regions for vesicle formation [46]. At the PAS, the isolation membrane develops and further expands into a double-membrane vesicle, an autophagosome. The autophagosome is subsequently delivered along microtubules to a lysosome, with which the outer membrane of the autophagosome fuses to form a so-called autolysosome. In this vesicle, the entrapped cargo is degraded by lysosomal enzymes and the process of autophagy is completed. The particular mechanisms of autophagy have been extensively reviewed elsewhere [47]. 

Importantly, autophagy may act as a double-edged sword when considered as a therapeutic target in cancer therapies. On the one hand, autophagy acts as a tumor suppressor by degrading potentially oncogenic molecules, which happens mainly at the beginning of tumorigenesis, on the other hand, autophagy strongly contributes to tumor cell survival and progression of tumor growth in its later stages. Tumor suppressor activity has been identified by particular ATGs. For example, Beclin 1 is monoallelically deleted in 40–70% of sporadic human breast, ovarian, and prostate cancers [48], mutations or deletion of *ATG5*, then, may lead to gastric, colorectal, or liver malignancies [49]. A possible antitumor effect of autophagy may lead to preventing necrosis and, thereby, tumorigenic inflammation in solid tumors [50]. The same effect is reached by autophagy-mediated senescence of cancer cells, which therefore do not proliferate rapidly, though they survive longer [51]. Cancer cell survival, even enhanced proliferation, however, may be the other side of autophagy regarding its roles in cancer progression. Such an effect could be caused, for example, by promoting glycolysis, as recently described in hepatocellular carcinoma cells [52]. Besides glucose metabolism, amino acid metabolism is also impacted by autophagy, which has been described as supplying human breast cancer cells with these essential catabolic molecules during nutrient deprivation [53]. In general, autophagy is used by cells as an adaptive response to stress. In cancer cells, autophagy leads to metabolic reprogramming, it supplies these cells with almost any biomolecules needed and speeds up their growth. However, predicting the exact effect of autophagy induction or inhibition on cancer progression during anticancer therapies is rather unpredictable, since there is another aspect, absolutely one of the most important ones, which must be considered. Namely, autophagy as a cell death modality [54]. 

**Figure 3:**
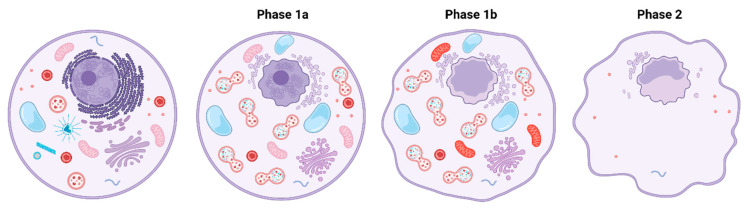
The process of autosis. In phase 1a, autosis can be recognized by initial vacuolization, endoplasmic reticulum dilation, and autolysosome formation. Phase 1b is characterized by the presence of electron-dense mitochondria and nuclear convolution. The perinuclear space is expanded and its focal swelling is mostly observed in phase 2 of autosis. In this phase, cellular organelles disappear and the endoplasmic reticulum is depleted. Created with BioRender.com (accessed on 4 November 2021).

Cell death is a tightly regulated process, which occurs in different forms [55]. Autophagy, together with apoptosis and necrosis, belongs to the three most often encountered types of cell death. The description of autophagic cell death is, however, rather complicated since the borders between cell death with autophagy and autophagy-driven cell death are inconsistent. The Nomenclature Committee on Cell Death defines an autophagy-dependent cell death as “a type of regulated cell death that relies on the autophagic machinery or components thereof”. This means that cell death is directly caused by autophagy and no other type of cell death is involved. Originally, autophagic cell death was characterized mainly by the autophagic vacuolization of the cytoplasm and the absence of chromatin condensation [56]. However, there are also other features connected with autophagic cell death, some of which have even led to a definition of an independent cell death type called autosis (Figure 3), in which a vital role is played by NKA. The term autosis was introduced by Liu et al. [57] in 2013 and it represents a unique mode of cell death characterized not only by an increase in autophagosomes, but also by ER dilation followed by later depletion, early-stage nuclear convolution, and late-stage focal swelling of the perinuclear space. Liu et al. confirmed that this type of cell death, which is caused by autophagy-inducing peptide Tat-Beclin 1 cannot be rescued by apoptosis or necroptosis inhibitors, however, it can be inhibited by CGs. Thus, interestingly, NKA is the key enzyme in autosis progression. This has been demonstrated also by the genetic knockdown of the NKA α1 subunit, which leads to complete inhibition of peptide- and starvation-induced autophagy in vitro. Autosis inhibition is, in addition to the inotropic effects of CGs, one of the reasons for its effect in rescuing heart injuries. Autophagy and autosis are often present in ischemia/reperfusion (I/R) injury of cardiomyocytes and cause the death of these cells [58]. By inhibiting NKA, CGs can rescue autosis-mediated cell death [59]. So far, the exact mechanisms of NKA involvement in autosis remain unresolved, however, some theoretical conclusions can be made from the already revealed NKA actions. For instance, CGs regulate the activity of nuclear factor kappa-light-chain-enhancer of activated B cells (NF-κB), which is a key transcription factor involved in sundry signal transduction pathways. The NF-κB activity can be either decreased [60,61] or increased [40,62,63] by CGs. The increased NF-κB activity is responsible for B-cell lymphoma 2 (Bcl2) protein activation subsequently leading to Beclin 1 inhibition. This might be an explanation for why some CGs rather inhibit autophagy or autosis instead of inducing it. The inhibition of autophagy by CGs occurs predominantly under starvation conditions. In contrast, under nutrient-rich conditions, CGs act as activators of the mTOR signaling pathway, which leads to autophagy progression [64]. Both induction and activation of autophagy by CGs lead to multifunctional impact on cell death since autophagy can be further linked to other types of cell death.

Much evidence has been published on the autophagy connection with other various cell death modalities. Examples of such cooperative action are autophagy-triggered ferroptosis [65], or necroptosis [66]. Besides, autophagy also triggers immunogenic cell death (ICD), i.e., the only cell death modality in which the immune system is activated. ICD is characterized by the release of damage-associated molecular patterns (DAMPs) such as high mobility group box 1, ATP, calreticulin, and many others from cells, which are exposed to ICD-inducing stress. Such molecules are then recognized by specialized components of the immune system. Thereby, ICD-inducing therapies may elicit a long-term anticancer effect and are, thus, extensively studied [67]. ICD progression is affected by the autophagy-supported release of ATP from dying cells as reported by Michaud et al. [68], who showed that, in *N*-nitroso-*N*-methylurethane-induced undifferentiated cells from colon carcinoma (CT26), the mitoxantrone-induced ATP release is inhibited upon autophagy suppression (*ATG5* and *ATG7* genes are deficient in CT26 cells). Thus, for cancer treatment, it would be worthy to discover/develop such compounds, which would have both autophagy- and ICD-potentiating properties. Such could be CGs, which exhibit induction of both of these cell death types. This makes CGs highly potent anticancer drug candidates [69]. In addition, it is not only autophagy and ICD that contribute to the overall antiproliferative action of CGs. The complexity of the CG mechanism of action is even greater since autophagy and ICD is also connected with necroptosis [70]. In addition to these types of cell death, autophagy also interacts with apoptosis [71,72] and many CGs can trigger both of these cell death modalities at once, as will be discussed further.

## 4. Cardiac Glycosides as Autophagy Modulators

As aforementioned, modulation of autophagy may be a double-edged sword. Therefore, treatment with CGs is also unpredictable. CGs have been described as both inducing and suppressing autophagy depending on the cell type being treated as well as on the particular CG. Therefore, understanding the CGs’ mechanisms of action is rather complicated and the results of CG treatment both in vitro and in vivo may vary. However, sundry studies have been reported on the great potential of CGs in anticancer therapy, but based on their ability to modulate autophagy, they have been also used in the treatment of other diseases. In the following sections, current knowledge on such activity of CGs will be discussed with a focus on their molecular mechanisms and clinical perspectives.

### 4.1. Bufalin

**Figure 4:**
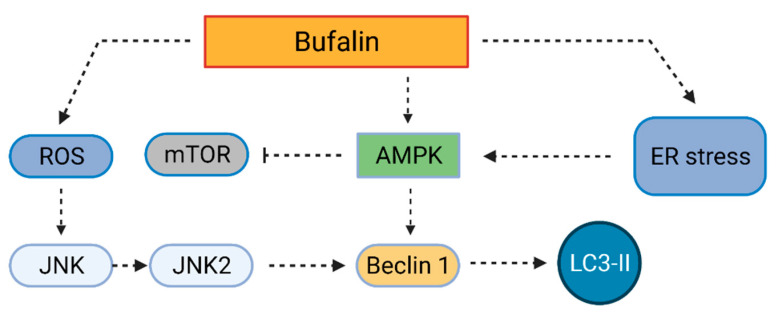
Mechanism of bufalin-induced autophagy. Bufalin treatment leads to reactive oxygen species (ROS) production and subsequent activation of c-Jun N-terminal kinase (JNK). This impacts the production of Beclin 1 and microtubule-associated protein 1A/1B-light chain 3-II (LC3-II). Beclin 1 and LC3-II are also produced in cells as a consequence of bufalin-induced adenosine monophosphate-activated protein kinase (AMPK) activation. AMPK activity is also impacted by endoplasmic reticulum stress, which can be also caused by bufalin. Created with BioRender.com (accessed on 23 November 2021).

Bufalin is a bufadienolide, which was originally isolated from the traditional Chinese medicine Chansu. It is present in the venom of the Asiatic toad *Bufo gargazians* and exerts various biological effects linked with NKA inhibition [73]. Its anticancer activity has been well characterized and an injectable form of Chansu, huachansu, has even been approved for cancer treatment by the China Food and Drug Administration [74]. The first study reporting on bufalin’s effect on autophagy has been published by Xie et al. [75] in 2011. They reported that in human cells derived from colorectal carcinoma (HT-29, and Caco-2), bufalin does not induce apoptosis at 100 nM concentration after 24 h, however, it significantly impairs cell survival, arrests cell cycle in the G2/M phase, and causes autophagic cell death. Autophagy, evidenced by an increase in the levels of microtubule-associated protein 1A/1B-light chain 3-II (LC3-II) in HT-29 cells is triggered by bufalin (100 nM, 48 h). The mechanism by which bufalin induces autophagy (Figure 4) in HT-29 cells has been attributed to ROS production and activation of c-Jun N-terminal kinases (JNK). The JNK-signaling pathway plays a role in Beclin 1 expression and is, thus, crucial in autophagy progression [76]. Xie et al. [75] reported that bufalin (100 nM) induces an increase in the levels of the active JNK2 form in a time-dependent manner (highest levels after 48-h treatment). This bufalin effect is probably dependent on ROS since inhibition of the ROS formation by *N*-acetylcysteine (10 mM) leads to a decrease in phosphorylated JNK2 levels in HT-29 cells treated with bufalin (100 nM, 24 h). To sum up, in colon cancer cells, bufalin induces cell death by increasing ROS formation and inducing autophagy without signs of apoptosis.

Similarly, in human cells from hepatocellular carcinoma (Huh7, Hep3B, and HA22T), Hsu et al. [77] confirmed that bufalin arrests cell cycle in the G2/M phase and induces autophagy by activating the JNK pathway. Bufalin half-maximal inhibitory concentrations (IC_50_) were reported to be 0.034–0.04 µM for each cell line after 72-h treatment. A qRT-PCR array analysis showed that several autophagy-related genes are upregulated in Huh7 cells by 0.04-µM bufalin treatment for 12 h. The most significantly increased expression (11.63-fold) was reported for gene encoding chemokine (C-X-C motif) receptor 4, which is a co-regulator of autophagy and apoptosis. Also, tumor necrosis factor is upregulated at the same conditions, which probably leads to the activation of the JNK-pathway, Beclin 1 production, and autophagy progression. Bufalin-induced autophagy further leads to apoptosis induction. As shown by Miao et al. [78], in human cells from hepatocellular carcinoma (HepG2), 100 nM bufalin induces apoptosis in 35.6 ± 8.4% of cells after 48 h of treatment. Simultaneously, bufalin induces autophagy in HepG2 cells, which is documented by increased Beclin 1 and LC3-II production. The fact that autophagy is a primary cause of apoptosis progression has been evidenced by co-treatment with 1 mM of autophagy inhibitor 3-methyladenine (3-MA). The 3-MA significantly decreases the percentage of apoptotic HepG2 cells after 48-h treatment with 100 nM bufalin from 35.6 ± 8.4% (without 3-MA) to 14.2 ± 6.3% (with 3-MA). Autophagy is in bufalin-treated HepG2 cells triggered via adenosine monophosphate-activated protein kinase (AMPK)-induced inhibition of mTOR. When pretreated with an AMPK inhibitor, compound C (1 µM), bufalin-induced upregulation of autophagy-related proteins is inhibited. Also, phosphorylation of mTOR is restored upon bufalin and compound C co-treatment. When bufalin is administered alone, the levels of phosphorylated mTOR significantly decrease with increasing concentration of bufalin, showing that mTOR inhibition is the basis for autophagy induction in HepG2 cells and, thereby, for apoptotic cell death in these cells.

In addition to hepatocellular carcinoma cells, the autophagy-apoptosis interplay has also been demonstrated in bufalin-treated glioma cells (U-87 MG, and LN-229). Shen et al. [79] reported that bufalin induces ER stress in these cells and, thereby, triggers apoptosis and autophagy. ER stress was documented by increased levels of CCAAT/enhancer-binding protein homologous protein (CHOP) and binding immunoglobulin protein (GRP78; BiP), as well as by increased phosphorylation of protein kinase R-like endoplasmic reticulum kinase (PERK) and eukaryotic translation initiation factor 2α (eIF2α). These effects occur in a dose-dependent manner after 24-h bufalin (tens of nM) treatment in both U-87 MG, and LN-229 cells. Interestingly, when ER stress is prevented (pretreatment with 500 µM tauroursodeoxycholate), the expression of LC3-II, as well as of other autophagy markers induced by bufalin, is reduced, showing that bufalin-induced ER stress precedes autophagy in glioma cells. The autophagy process, however, can be triggered by bufalin also via AMPK and mTOR signaling as described above. Importantly, Shen et al. showed that autophagy inhibition significantly decreases the viability of bufalin-treated cells. When bufalin (40 nM) is combined with an autophagy inhibitor 3-MA (3 mM), or wortmannin (1 µM), cell viability decreases by 20% in comparison with sole bufalin treatment for both 24 and 48 h. 

Thus, combining bufalin with autophagy inhibitors could be a good option in the development of anticancer regimens. This has been confirmed by Zhao et al. [80], who studied the effects of bufalin on human gastric cancer cells (SGC 7901, and BGC-823). They reported a strong inhibition of cancer cell proliferation by bufalin treatment (48 h, up to 200 nM), whereas gastric mucous epithelial cells (GES-1) were unaffected even at the highest concentration tested (200 nM). Bufalin (80 nM, 48 h), similar to the aforementioned studies, also induced ER stress-mediated autophagy and promoted apoptosis via the JNK pathway in both types of tested cells. When bufalin is combined with 10 mM 3-MA or when *ATG5* is silenced by a corresponding small interfering ribonucleic acid (siRNA), its antiproliferative effect increases. Besides gastric cancer cells, the same was also detected in liver cancer cells as reported by Sheng et al. [81]. In human cells from hepatocellular carcinoma (HCCLM3), the highest antiproliferative activity was observed in cells treated with bufalin (0.12 µg·mL^−1^) in combination with 3-MA (5 mM) and chloroquine (5 µg·mL^−1^) for 24 h. The inhibition rate, in this case, was 20% higher than was achieved with sole bufalin treatment. This clearly shows that in some cell types, in particular in cells from hepatocellular carcinoma, glioma, and gastric carcinoma, autophagy caused by bufalin is rather protective and, thus, it is worthy to combine bufalin with autophagy inhibitors to increase its antiproliferative activity. 

### 4.2. Digoxin

In addition to bufalin, another CG, digoxin, has been found to induce autophagy. Digoxin is a cardiotonic compound, which was first isolated by S. Smith in 1930 from *Digitalis lanata*, the Balkan foxglove [82]. Currently, it is widely used in cardiac medicine, mainly against heart failure and arrhythmias [83], however, its anticancer potential is emerging in clinical trials and the mechanisms of its anticancer actions have been studied extensively. A great role in these mechanisms is played by autophagy. The first study to report autophagy induction by digoxin was published by Hundeshagen et al. [84], who performed a flow cytometry-based screening of autophagy inducers. They identified digoxin, together with its aglycone digoxigenin and strophanthin, as a potent autophagy inducer. In human cells from breast carcinoma (MCF-7), digoxin induced both autolysosome formation and degradation at concentrations ranging from 10 ng·mL^−1^ to 10 μg·mL^−1^, at which it significantly upregulates lysosomal turnover (loss of fluorescence emission detection of Rab7 [Ras-related protein] co-expressed with the green fluorescent protein). Importantly, digitoxin maximally impacts the functions of autolysosomes at 100 ng·mL^−1^ concentration, which is a concentration present in the blood plasma of cardiac patients and, thus, is safe and useful in further preclinical and potential clinical trials. Before, however, the mechanisms of digoxin action must be elucidated. 

**Figure 5:**
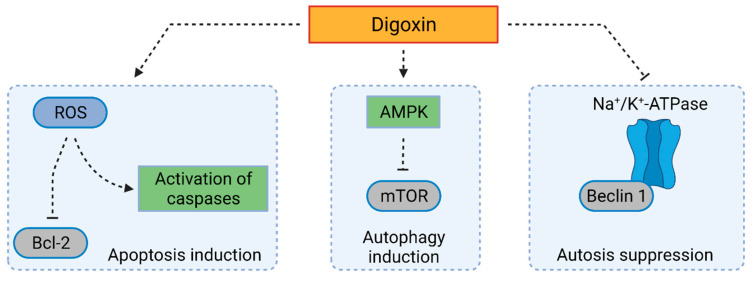
Mechanism of digoxin-induced autophagy and apoptosis. Digoxin treatment leads to reactive oxygen species (ROS) production and subsequent inhibition of B-cell lymphoma-2 (Bcl2) protein and activation of caspases, which means activation of apoptosis. At the same time, digoxin induces autophagy via adenosine monophosphate-activated protein kinase (AMPK) activation and inhibition of the mammalian target of rapamycin (mTOR). Digoxin also suppresses autophagy by preventing the binding of Beclin 1 to Na^+^/K^+^-ATPase. Created with BioRender.com (accessed on 23 November 2021).

Unfortunately, Hundeshagen et al. did not study any particular mechanisms of digoxin-induced autophagy. At that time, the authors attributed this activity solely to the digoxin’s ability to disrupt the Ca^2+^ balance inside cells [84]. More recently, the ambiguous role of Ca^2+^ ions has been described in connection to autophagy. Ca^2+^ ions can both induce and suppress autophagy and they are also an important link between autophagy and apoptosis, which only contributes to the discussed complexity of CG-autophagy interplay [85]. Also, digoxin has recently been described as inducing both autophagy and apoptosis (Figure 5). In the study by Rasheduzzaman et al. [86] digoxin is shown to activate caspases in hepatocellular carcinoma resistant to tumor necrosis factor-related apoptosis-inducing ligand (TRAIL). This caspase activation occurred via ROS production, downregulation of the expression of anti-apoptotic Bcl2 protein, and increased translocation of a proapoptotic protein—cytochrome c. However, digoxin simultaneously induces protective autophagy via AMPK-pathway, which significantly decreases the probability of cell death. In particular, when Huh-7 and HepG2 cells are treated with digoxin (up to 100 nM) for 16 h, cell viability is not significantly affected in comparison with an unaffected control. However, when TRAIL is added for 4 h to digoxin (100 nM) treatment (12 h), cell viability decreases by 50%. Cell viability inhibition rates can be significantly decreased by ROS scavengers, such as *N*-acetylcysteine. When both cell types are pretreated with *N*-acetylcysteine (15 mM) for 1 h, cell viability increases from 50% to 80% in comparison with the untreated control, which points to the essential role of ROS in the antiproliferative action of digoxin. ROS production can be enhanced by the inhibition of autophagy, which leads to decreased cell viability. In particular, co-treatment with an autophagy inhibitor chloroquine (20 µM) leads to a significant increase in ROS levels (measured by flow cytometry using 2′,7′-dichlorodihydrofluorescein diacetate) and corresponding inhibition of Huh-7 and HepG2 cell viability. 

However, autophagy induction by digoxin, might not always be a big concern in anticancer therapy. A recent study by Crezee et al. [87] shows digoxin as a potential treatment option for non-medullary thyroid cancer (NMTC) patients. In the cohort of patients suffering from NMTC with cell dedifferentiation, the classic therapy with radioactive iodine is not successful, since dedifferentiated cells are not able to accumulate the drug. In TPO-Cre/LSL-Braf^V600E^ transgenic mice, digoxin (20 and 60 µM) inhibited tumor growth in comparison with vehicle-treated control at days 5, 12, and 19 after administration. The tumor volume of digoxin-treated mice remained the same as before the treatment, whereas, in the vehicle-treated cohort, the tumor growth continued, and the tumor volume increased from 25 to 40 mm^3^ at day 20. Digoxin (20 µM) also supports ^124^I uptake into tumor cells (a 60% increase compared with vehicle-treated control). This could already be observed on day 5 of digoxin treatment. For both digoxin concentrations, a 20% increase in autophagy activity was detected. Importantly, in a retrospective clinical study of NMTC patients simultaneously treated with digoxin, this compound is shown to provide better clinical outcomes for the selected patients. This is in agreement with the provided observations; digoxin induces autophagy, sensitizes NMTC cells to ^124^I, and also induces expression of various thyroid-specific genes and thereby increases thyroid cell differentiation (12.8-fold higher thyroid differentiation score in comparison with untreated control after 20-µM digoxin treatment). 

However, in contrast to the aforementioned studies, digoxin has been reported also to suppress autophagy, and in particular, to suppress autosis. In human cells derived from cervical carcinoma (HeLa) and osteosarcoma (U-2 OS), digoxin suppresses autosis at IC_50_ values below 0.1 μM. Liu et al. induced autophagic cell death with the Tat-Beclin 1 peptide and observed its unique morphological appearance described above. These morphological changes during Tat-Beclin 1-induced autosis can be reversed by digoxin, except for the presence of electron-dense mitochondria. This phenomenon, however, can also be observed in cells treated only with digoxin and not with Tat-Beclin 1. Recently, more insight into the autosis inhibition mechanism has been presented by Fernández et al. [88]. In starved or stressed cells and tissues, such as livers of patients with anorexia nervosa, brains of neonatal rats subjected to cerebral hypoxia/ischemia, and kidneys of mice subjected to renal I/R injury, NKA interacts with Beclin-1. Autophagy induced by starvation or Tat-Beclin 1 in HeLa cells leads to increased co-immunoprecipitation of NKA with Beclin 1. Similar results were obtained from proximity ligase assays. Interestingly, the interactions between NKA and Beclin 1 occur both in the cytoplasm and different intracellular compartments, i.e., ER, nuclear membrane, mitochondria, or early endosomes. Most importantly, Fernández et al. discussed the roles of both endogenous and exogenous CGs. They found out that endogenous digoxin-like molecules decrease the interaction between NKA and Beclin 1 since when mice were pretreated with an antibody against these molecules (DigiFab, 10 mg·kg^−1^), the interaction significantly increased after 80 min of exercise. Normally, though, autophagy is induced while exercising, however, presumably due to the action of digoxin-like endogenous factors, the interaction between NKA and Beclin 1 is not naturally increased. As for the exogenous CGs, the authors found that under the treatment with ouabain, the discussed protein-peptide interaction is decreased and, thus, ouabain prevents autosis. 

### 4.3. Ouabain

**Figure 6:**
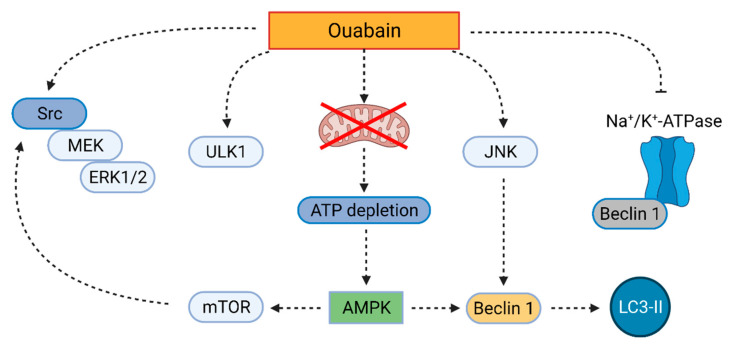
Mechanism of ouabain-induced autophagy. Ouabain induces autophagy via proto-oncogene tyrosine-protein kinase (Src)/mitogen-activated protein kinase MEK/extracellular signal-regulated kinases 1 and 2 (ERK1/2) pathway, Unc-51 like autophagy activating kinase (ULK-1), and c-Jun N-terminal kinase (JNK) activation. The latter leads to the production of Beclin 1 and microtubule-associated protein 1A/1B-light chain 3-II (LC3-II). Another autophagy-inducing pathway leads via inhibition of mitochondrial respiration, activation of adenosine monophosphate-activated protein kinase (AMPK), and subsequent activation of the mammalian target of rapamycin (mTOR), which activates the Src/MEK/ERK1/2 pathway. In addition, ouabain inhibits the binding of Beclin 1 to Na^+^/K^+^-ATPase and, thus, prevents autosis. Created with BioRender.com (accessed on 23 November 2021).

Ouabain is a cardenolide compound, which occurs naturally in plants, such as *Strophantus gratus* and *Acokanthera schimperi* [89]. For a long while, ouabain had been considered to also occur endogenously in humans, however, recent analyses confirm that the so-called “endogenous ouabain” is not identical with plant ouabain and occurs even in various isomers [90]. The plant ouabain was first identified to induce autophagy by Wang et al. [64] in 2012. They reported increased levels of LC3-II, ATG5, Beclin 1, and AMPK, as well as decreased levels of activated mTOR in human cells derived from non-small cell lung carcinoma (A549, and H460), treated with 25 nM ouabain. These changes were mostly visible after 24-h treatment. Autophagy induction by ouabain (Figure 6) is linked to the induction of extracellular signal-regulated kinases 1 and 2 (ERK1/2), since co-treatment, with 10 µM PD98059, an inhibitor of mitogen-activated protein kinase kinases 1 and 2 (MEK 1/2), significantly decreases LC3-II level simultaneously with decreased activation of ERK1/2. Wang et al. also presented the same results for 50 nM digoxin. In their further research, the authors confirmed the mechanism of action of both ouabain and digoxin in lung cancer cells and identified Src kinase as an important part of the ERK1/2 signaling cascade. The inhibitor of Src kinase, 10 µM PP2 (1-*tert*-Butyl-3-(4-chlorophenyl)-1*H*-pyrazolo[3,4-*d*]pyrimidin-4-amine), markedly decreases the levels of phosphorylated ERK and MEK and reverses the induction of autophagy by CGs, as well as increases the cell viability by 15%, when A549 and H460 cells are treated with either 25 nM ouabain or 50 nM digoxin [91]. Recently, the same research group further clarified the ouabain-induced autophagy mechanism in A549 cells. The Src pathway is linked to the AMPK/mTOR pathway, since the 6-h treatment with 10 µM compound C, an AMPK inhibitor significantly downregulates Src phosphorylation. However, inhibition of Src by 10 µM PP2 does not affect the level of phosphorylated AMPK, thus, in ouabain-treated A549 cells, AMPK acts as an upstream Src regulator. Moreover, 25 nM ouabain affects cancer cell metabolism and thereby the activation of AMPK. It disrupts mitochondrial respiration and causes significant ATP depletion (a 50 and 70% decrease after 2 and 24 h, respectively). AMPK is activated as a consequence of these changes. Thus, in lung cancer cells, the mechanism of ouabain-induced autophagy is based on metabolic changes, disrupted mitochondrial respiration leading to AMPK/mTOR pathway activation, and subsequent Src/MEK/ERK induction [92].

However, other pathways are also involved in ouabain-induced autophagy. Trenti et al. reported classical patterns of autophagy in A549, similarly to the aforementioned studies. Ouabain (100 nM) induced an increase in LC3-II level in a time-dependent manner and induced autophagic flux. In addition to the aforementioned characteristics, Trenti et al. [93] reported activation of Unc-51 like autophagy activating kinase (ULK1), which is involved in autophagy initiation. Ouabain (100 nM) induces l-Ser^555^ phosphorylation and l-Ser^757^ dephosphorylations in ULK1 after 24-h treatment of A549 cells, as confirmed by immunoblotting. Importantly, ouabain also induces rapid phosphorylation of JNK and 30 min pretreatment with a JNK inhibitor SP600125 (30 µM) significantly reverses cytotoxic activity of ouabain and reduces the markers of autophagy after 24 h of ouabain treatment. Thus, both ULK1 and JNK play an important role in ouabain-induced autophagy. Collectively, due to autophagy induction, ouabain could be a useful drug in the treatment of lung cancer. This also confirms the recent research of Rupaimoole et al. [94], who discussed the possible combinatorial use of ouabain with miRNA-34, which is a tumor suppressor miRNA often lost in tumors. The synergism of ouabain (30 nM) with this miRNA (40 nM) in A549 cells after 24 h is based on autophagy induction. The levels of LC3-II are higher when both drugs are used in combination. The exact mechanism of miRNA-34’s contribution to ouabain-induced autophagy in lung cancer cells, however, remains to be elucidated.

The autophagy induced by ouabain has been extensively studied and it might not only be useful in cancer treatment but also, for example, in the treatment of Alzheimer’s disease. The involvement of transcription factor EB (TFEB), a regulator of autophagy-related gene expression, in autophagy induction has been reported in ouabain-treated neural cells. Ouabain (500 nM) causes TFEB translocation from cytosol to nucleus after 8 h in HeLa cells and primary cortical neurons. TFEB is dephosphorylated and active after ouabain treatment and autophagy is triggered, something that is confirmed by increased levels of LC3-II by immunoblot. Most importantly, Song et al. [95] suggest that the effect of ouabain on TFEB activation may be useful in the treatment of Alzheimer’s disease. Activation of TFEB decreases the amount of phosphorylated tubulin-associated unit (Tau) protein, the levels of which correlate with the degree of dementia. It has been shown that ouabain decreases the levels of phopho-Tau both in vitro (human cells from neuroblastoma SH-SY5Y and primary neurons treated with 500 nM ouabain for 8 h) and in vivo (TauP301L mice with induced memory impairment and elevated tau level, behavioral tests after administration of 1.5 μg·kg^−1^ ouabain). Through mTOR-driven protective autophagy, ouabain induces cellular restorative properties in Alzheimer’s disease models.

Autophagy may in some cell types be combined also with apoptosis when treated with ouabain. In Burkitt’s lymphoma Raji cells both cell death types are triggered after 48 h of treatment with 100 nM ouabain. This is demonstrated by the presence of autophagosomes (microscopic analysis), increased levels of LC3-II, Beclin 1, cleaved caspase 3, or Bcl2-associated X protein, and decreased levels of Bcl2. These findings support the idea of using ouabain for the treatment of Burkitt’s lymphoma [96]. Ouabain, due to its autophagy-modulating properties, could be also useful in the treatment of heart injuries. Nah et al. [97] have reported that myocardial I/R induces autosis in cardiomyocytes and thereby even complicates the severe condition. In humanized NKA α1 knock-in mice, 0.56 mg·kg^−1^ ouabain treatment 3 and 6 h after 30 min of ischemia reduces the heart tissue injury. This happens due to the inhibition of autosis by ouabain. Similarly, Fernández et al. have reported that ouabain (0.25 mg·kg^−1^, peritoneal administration) can decrease autosis by inhibiting Beclin 1–NKA interaction in mouse kidneys with I/R injury [88]. In addition, ouabain could be useful also in therapeutic senolytic strategies, since, as recently reported by L’Hote et al. [98], autophagy sensitizes senescent cells to senolysis. Cells, in which senescence is induced by the expression of v-raf murine sarcoma viral oncogene homolog B1 (BRAF), were significantly more sensitive to ouabain treatment (complete inhibition of cell viability by 200 nM ouabain after 72 h) than proliferating BJ fibroblasts (no inhibition). This difference can probably be explained by the fact that BRAF-V600E, which is a hyperactive mutant of BRAF, causes chronic ER stress and autophagic flux in senescent cells. This chronic autophagy is inhibited by ouabain, which thereby acts as a senolytic compound. The aforementioned studies show that autophagy modulation might be a very useful mechanism in novel anticancer and other therapies and ouabain has, therefore, a great therapeutical potential. However, there are also many other CGs, which modulate autophagy.

### 4.4. Digitoxin

Digitoxin is also among the CGs which have been described to possess autophagy-modulating properties. Digitoxin is a cardenolide naturally occurring in *Digitalis* sp. Digitoxin, together with digoxin, has been reported to be potentially useful in the treatment of ovarian clear cell carcinoma (OCCC). Hsu et al. [99] identified an NKA subunit FXYD2 to be overexpressed in this type of cancer and described both CGs as suppressing its progression via autophagic cell death potentiation. The IC_50_ of digitoxin for TOV-21G, IGROV-1 (OCCC cell lines), and SK-OV-3, A2780 (non-OCCC) cell lines are 2, 1, 68, and 84 nM respectively. Interestingly, significantly higher concentrations are needed for inhibition of non-OCCC cell viability, which indicates that the FXYD2 subunit is important in the digitoxin mechanism of action. Five nM digitoxin induces autophagy in TOV-21G cells after 48 h, as confirmed by fluorescence microscopy of LC3-II fused with a green fluorescent protein. Digitoxin, thus, may be beneficial against the specific cancer type, OCCC. Moreover, digitoxin, due to its autophagy-inducing property, could be useful in antiviral therapy. Various CGs have been described as possessing antiviral activity [100]. Digitoxin suppresses human cytomegalovirus (HCMV) replication via induction of the AMPK pathway and autophagy. In human foreskin fibroblasts, 30 nM digitoxin induces AMPK phosphorylation after 12 h of treatment, and this activation persists during infection for 72 h. The maximal antiviral activity, however, can be observed in the first 24 h after digitoxin treatment. Besides AMPK activation, 30 nM digitoxin induces ULK1 phosphorylation on ser317 after 24 h (HCMV infection leads to l -Ser^757^ phosphorylation and autophagy suppression) and suppresses mTOR activation (HCMV induces it) in cells with compromised autophagy (knockdown of ATG5), digitoxin then loses the antiviral activity [101].

### 4.5. Lanatoside C, Strophantidin, Peruvoside, Convallatoxin

**Figure 7:**
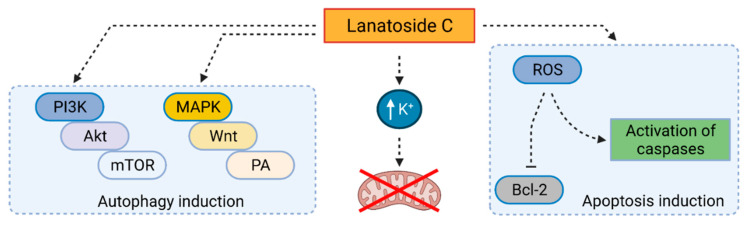
Mechanism of lanatoside C-induced autophagy. Lanatoside C induces autophagy by triggering phosphatidylinositol 3 kinase (PI3K)/protein kinase B (Akt)/mammalian target of rapamycin (mTOR) pathway or by activating mitogen-activated protein kinase (MAPK)/Wingless-related integration site protein (Wnt)/palmitic acid (PA) pathway. Autophagy may also be triggered as a consequence of mitochondrial activity inhibition due to the increased intracellular level of K^+^ by lanatoside C. Together with autophagy, lanatoside C also triggers apoptosis, in particular via reactive oxygen species (ROS) production, inhibition of B-cell lymphoma protein 2, and activation of caspases. Created with BioRender.com (accessed on 23 November 2021).

Another CG-modulating autophagy is lanatoside C, a cardenolide compound from *Digitalis lanata*. Kang et al. [102] have described lanatoside C as a potent inhibitor of colorectal cancer cell growth and observed classical patterns of autophagy in the treated cells. In human cells from colorectal carcinoma (HCT 116), LC3-II levels were affected by the CG directly proportional in time- and dose-dependent manner (24 or 48 h, max. 1 µM concentration). The mechanism of action (Figure 7) is linked to phosphorylation of ERK1/2 and JNK (mostly visible at 1 µM concentration of lanatoside C after 4 h). The CG also causes mitochondrial dysfunction by decreasing the intracellular level of K^+^, which can be demonstrated, for example, by morphological changes observed microscopically. More recently, lanatoside C was shown to inhibit hepatocellular carcinoma by inducing autophagy. Its effects against TRAIL-resistant Huh7 and HepG2 cells are similar to those of digoxin which are described above [86]. The anticancer mechanisms of lanatoside C have further been confirmed by Reddy et al. [103]. In A549, MCF-7, and HepG2 cells, lanatoside C arrests cells in the G2/M phase by blocking the MAPK/Wingless-related integration site protein/palmitic acid pathway and induces apoptosis by blocking autophagic pathway PI3K/Akt/mTOR at concentrations 0.16, 1.2, and 0.7 µM for A549, MCF-7, and HepG2 cells, respectively, after 24 h of treatment. Thus, in this case, the benefit of CG is in autophagy inhibition and not induction.

Besides lanatoside C, Reddy et al. also identified more CGs as autophagy modulators. One such example is strophanthidin, which had been found already in the screening study of Hundeshagen et al. [84]. Reddy et al. [104] described this cardenolide occurring in *Strophantus* sp. to have very similar properties as lanatoside C on A549, MCF-7, and HepG2 cells, i.e., it inhibits autophagy and triggers apoptosis in the studied cells at a concentration of 1, 2, and 2,5 µM for A549, MCF-7, and HepG2 cells respectively after 24 h. Another plant cardenolide, peruvoside, which occurs naturally in *Cascabela thevetia*, also inhibits autophagy via suppression of the PI3K/Akt/mTOR pathway and also by blocking the MEK1 pathway in the same cells (100 nM, 24 h) [105]. Peruvoside was also studied by Kaushik et al. [106], who confirmed its anticancer properties on human lung cancer cells and tumorspheres. Peruvoside induces phosphorylation of ERK, MAPK p38, and Akt, which suggests autophagy induction. Interestingly, another CG, convallatoxin from *Convallaria* sp., which has been studied together with peruvoside, exerts an opposite action and rather decreases the activation of Akt and MAPK p38. This suggests that a great role in the mechanism of action of both CGs is played by the sugar moiety, which represents the only difference between both compounds. Convallatoxin has been studied more deeply by Yang et al. [107], who identified this compound (isolated from *Antiaris toxicaria*) as an inducer of both apoptosis and autophagy. They tested convallatoxin on a panel of human cancer cell lines and reported potent inhibitory activity demonstrated by very low IC_50_s between 4 and 40 nM after 72 h. In HeLa cells, convallatoxin (10 nM) induces apoptosis as shown by increased caspase-3 and poly (adenosine diphosphate-ribose) polymerase (PARP) cleavage. This action is time-dependent, the highest cleaved PARP ratio can be observed after 48 h. Autophagy, in particular LC3 conversion, occurs mostly after 24 h of treatment with 10 nM convallatoxin, which in the same amount and over the same period also causes inhibition of mTOR and ribosomal protein S6 kinase beta-1 phosphorylation. Importantly, Yang et al. also identified convallatoxin as a useful tool for angiogenesis inhibition both in vitro and in vivo. In vitro, convallatoxin inhibits human umbilical vein endothelial cell tube formation at 4 nM concentration after 16 h of treatment; in vivo, it inhibits angiogenesis in chicken eggs (0.5 ng per egg, 48 h of treatment). This activity of convallatoxin is based on autophagy induction since inhibition of autophagy by *ATG5* knockdown (with use of siRNA) significantly reduces the antiangiogenic action of convallatoxin.

### 4.6. Oleandrin, Divaricoside, Proscillaridin A, Glucoevatromonoside, Neriifolin

Many other plant cardenolides have been shown to possess autophagy-modulating properties. One such is oleandrin, which can be isolated from *Nerium oleander*. This CG arrests the cell cycle in the G2/M phase and causes autophagic cell death in human cells from pancreatic carcinoma (PANC-1). Oleandrin (20 nM) causes increased levels of LC3-II after 72 h, 50 nM of the compound then increases ERK phosphorylation and decreases Akt phosphorylation after 24 h, which clearly shows autophagy induction [108]. A recent study of Terzioglu-Usak et al. [109] confirms the autophagy-modulating property of oleandrin in commercially available form, Anvirzel^TM^, which consists of oleandrin and oleandrigenin. Anvirzel^TM^ modulates the Akt/mTOR pathway, activates ERK and mTOR phosphorylation, decreases Akt and JNK1/2 phosphorylation in human glioblastoma cells U-87 MG after 48 h. The formulation was tested in various concentrations from 10 to 250 µg·mL^−1^ and in immunoblot analysis, the levels of particular proteins peaked by different concentrations. In general, however, the higher concentration of Anvirzel^TM^, the greater the autophagy induction. Autophagy is also induced by another plant cardenolide, divaricoside, which is isolated from *Strophanthus divaricatus*. This compound inhibits the growth of cells derived from oral squamous carcinoma (SCC2095) by autophagy induction observed as LC3-II and p62 upregulation (10 to 500 nM, 48 h). At the same time, it induces apoptosis by triggering pathways connected with myeloid-cell leukemia 1 (Mcl-1) protein from the Bcl2 family of proteins. In general, Bcl2 protein family levels are significantly affected by divaricoside (downregulation of Mcl-1, Bcl2, upregulation of Bcl2 associated X protein and phorbol-12-myristate-13-acetate-induced protein 1 in a dose-dependent manner from 10 to 500 nM, 48 h) [110].

In addition to cardenolides, of course, bufadienolides have also been described as autophagy inducers. One such example, besides the aforementioned bufalin, is proscillaridin A, a compound occurring in *Scilla* sp. In hepatocellular carcinoma cell lines, proscillaridin A (10 to 500 nM, 48 h) causes mitochondrial damage as observed by transmission microscopy, which happens due to ROS formation. Fifty nM of proscillaridin A induces autophagy in human cells derived from hepatocarcinoma (MHCC97H, and Huh7) after 24 h of treatment (LC3-II increase, inhibition of mTOR) [111]. As confirmed by Saleem et al. [112], proscillaridin A could be combined with autophagy inhibitors to increase its anticancer potential. In breast cancer, proscillaridin A induces both apoptosis and autophagy. In human cells from breast carcinoma (MCF-7, and MDA-MB-231), Apoptosis is triggered via DNA fragmentation, caspase cascade activation, PARP cleavage, etc.; autophagy is evidenced by LC3 conversion, JNK activation, and decreased phosphorylation of mTOR and Akt (up to 100 nM concentrations, 24 h). When co-treated with JNK inhibitor SP600125 (20 µM, 8 h), apoptosis markers significantly increase and, thus, cells are more likely to die under the treatment with proscillaridin A.

As shown in proscillaridin A treatment, inhibition of autophagy might in some cases be beneficial for achieving better anticancer activity. Some CGs can inhibit autophagy themselves. One of such is glucoevatromonoside, which occurs naturally in *Digitalis* sp. It has been described as potently inhibiting autophagy in A549 cells at 50 nM concentration after 24 h treatment. It causes reduction of LC3 conversion, decreased levels of Beclin 1, and autophagy receptor p62 in 10 nM bafilomycin-treated cells [113]. Another example of autophagy-inhibiting CGs is neriifolin. This compound was identified as an autosis inhibitor by Liu et al. [57], who performed a chemical screen of ∼5000 bioactive compounds for anti-autosis activity and identified various CGs as being capable of such action. Due to this ability, neriifolin is highly neuroprotective in rats, in particular, it reduces neonatal hypoxia/ischemia brain damage. It increases the intact area of the hippocampus from 20% (treatment only with the vehicle—0.5% ethanol/phosphate buffer) to 80% (0.25 mg·kg^−1^ neriifolin injected intraperitoneally immediately after carotid artery occlusion). This correlated clearly with autophagy markers since, in the vehicle-treated cohort, LC3 conversion occurred significantly more than in the neriifolin group. The usefulness of neriifolin in I/R injury treatment has been confirmed also by Fernández et al., who described neriifolin (0.22 mg·kg^−1^, intraperitonally) to inhibit Beclin 1–NKA interaction and autosis in the hippocampal region of rat pups [88]. Neriifolin could be useful also in the treatment of autoimmune encephalomyelitis. Keller et al. [114] reported neriifolin (0.25 mg·kg^−1^) as inhibiting ATG-dependent phagocytosis in vivo, which delays onset (5 days delay in comparison with vehicle control) and reduces the clinical severity of experimental autoimmune encephalomyelitis. In mouse macrophages (RAW 264.7), neriifolin (1 µM, 24 h) potently inhibits autophagy (decrease in LC3-II). Although certain insight into the mechanisms of autosis inhibition by CGs has been published already (discussed above in the digoxin section) [88], the exact interactions between the autophagy machinery in different cell types with different CGs remains to be understood. CGs are worth further study in general, and not only for their autophagy modulating properties (Table 1)

## 5. Conclusions

CGs, drugs used for heart failure and arrhythmia treatment, are emerging anticancer drug candidates. The basis of CGs’ antiproliferative properties is, besides apoptosis, autophagy modulation. CGs act specifically in different cell types; they can both suppress and induce autophagy. Suppressive activity has been reported mainly against autosis, an NKA-dependent autophagy type, which is in most cases cytoprotective. The induction of autophagy and autophagic cell death then occurs as a consequence of AMPK/mTOR pathway potentiation with subsequent induction of the Src/MEK/ERK pathway. However, other mechanisms also contribute to CG-induced autophagy, namely ROS production and mitochondrial damage, TFEB translocation, or JNK pathway modulation. The usefulness of autophagy induction by CGs is, however, cell-type-dependent. Whereas in some types of cancer cells, such as cells from lung carcinoma, the autophagic cell death is beneficial in the treatment, in other cell types, such as ones derived from gastric and hepatocarcinoma, combination with autophagy inhibitors increases the antiproliferative properties of CGs. Therefore, the potential involvement of CGs in anticancer therapies must also be thoroughly considered. Despite many published studies revealing the autophagy-modulating actions of CGs, which are summarized in this article, there is still a lack of knowledge about the mechanisms and reasons for the fully different actions of particular CGs in particular cancer cell types. Even, in addition to anticancer properties, studies have been published on the potential use of CGs against Alzheimer’s disease or viral infections, both as a result of autophagy inhibition. This evidence complicates our understanding of autophagy modulation by CGs and its consequences even more. Because autophagy modulation contributes to the great clinical potential of CGs in anticancer and other therapies, it is of high importance to study this property of CGs extensively. Only an understanding of the mechanisms which stand behind the autophagy-modulating activity of CGs can move the clinical success of CGs further.

## Figures and Tables

**Table 1:** Induction or suppression of autophagy by cardiac glycosides and modulation of autophagy-related molecular markers.

Cardiac Glycoside	Cell Line	Concentration, Incubation Time	Marker	Autophagy Induced (+)/ Suppressed (−)	Ref.
Bufalin	HT-29	100 nM, 48 h	↑ LC3-II, ↑ ROS, ↑ JNK2	+	[75]
	Huh7	40 nM, 12 h	↑ chemokine receptor 4, ↑ tumor necrosis factor, ↑ JNK, ↑ Beclin 1	+	[77]
	HepG2	100 nM, 48 h	↑ pAMPK, ↓ mTOR, ↑ LC3-II, ↑ Beclin 1	+	[78]
	U87MG	20–80 nM, 24 h	↑ CHOP, ↑ GRP78, ↑ pPERK, ↑ p (eIF2α)	+	[79]
	LN229			+	
	SGC7901	80 nM, 48 h	↓ LC3-I, ↑LC3-II, ↓ p62	+	[80]
	BGC823			+	
Digoxin	MCF-7	100 ng/mL, 6 h	↑ lysosomal turnover	+	[84]
	Huh7	>100 nM, 16 h	↑ pAMPK	+	[86]
	HeLa	>10 nM, 24 h	↑LC3-II, ↓ p62	-	[57]
	A549	50 nM, 24 h	↑ LC3-II,↑ ATG5, ↑ AMPK, ↓ mTOR	+	[64,91]
	H460			+	
Ouabain	A549	25 nM, 24 h	↑ LC3-II,↑ ATG5, ↑ AMPK, ↑ Beclin 1, ↓ mTOR	+	[64,91,92]
	H460			+	
	A549	100 nM, 24 h	↑ LC3-II, ↑ pULK1, ↑ pJNK	+	[93]
	HeLa	500 nM, 8 h	↑ LC3-II, ↑ TFEB	+	[95]
	Raji	100 nM, 48 h	↑ LC3-II, ↑ Beclin 1, ↓ caspase 3, ↓ Bcl2	+	[96]
Digitoxin	TOV-21G	5 nM, 48 h	↑ LC3-II	+	[99]
	HFF	30 nM, 24 h	↑ pAMPK, ↑ pULK1, ↓ mTOR	+	[101]
Lanatoside C	HCT116	1 μM, 4 h	↑ LC3-II, ↑pJNK, ↑ pERK 1/2	+	[102]
	A549	160 nM, 24 h	↓ Akt, ↓ mTOR, ↓PI3K, ↓ LC3	-	[103]
	MCF-7	1.2 μM, 24 h		-	
	HepG2	700 nM, 24 h		-	
	Huh7	>100 nM, 16 h	↑ pAMPK	+	[86]
Strophanthidin	A549	1 μM, 24 h	↓ Akt, ↓ mTOR, ↓PI3K, ↓ LC3	-	[104]
	MCF-7	2 μM, 24 h		-	
	HepG2	2.5 μM, 24 h		-	
Peruvoside	A549	100 nM, 24 h	↓ Akt, ↓ mTOR, ↓PI3K, ↓ LC3, MEK1 blocking	-	[105]
	MCF-7			-	
	HepG2			-	
	H460	25 nM, 24 h	↑ pERK, ↑ pMAPK p38, ↑ pAkt	-	[106]
Convallatoxin	H460	25 nM, 24h	↓ pERK, ↓ pMAPK p38	-	
	HeLa	10 nM, 24 h	↑ LC3-II, ↑ p (S6 kinase beta-1), ↓ mTOR	+	[107]
Oleandrin	PANC-1	20 nM, 72 h	↑ LC3-II	+	[108]
	PANC-1	50 nM, 24 h	↑ pERK, ↓ pAkt	+	
Anvirzel^TM^	U87	10–250 µg/mL, 48 h	↑ pERK, ↑ p(mTOR), ↓ pAkt, ↓ pJNK1/2	+	[109]
Divaricoside	SCC2095	10–500 nM, 48 h	↑ LC3-II, ↑ p62	+	[110]
Proscillaridin A	MHCC97H	50 nM, 24 h	↑ LC3-II, ↓ mTOR	+	[111]
	Huh7			+	
	MCF-7	>100 nM, 24 h	↑ LC3-II, ↑pJNK, ↓ p (mTOR), ↓ pAkt	+	[112]
	MDA-MB-231			+	
Glucoevatromonoside	A549	50 nM, 24 h	↓ LC3-II, ↑LC3-I, ↓ p62, ↓Beclin 1	-	[113]
Neriifolin	RAW 264.7	1 µM, 24 h	↓ LC3-II	-	[114]

↑ increased levels of the protein, ↓ decreased levels of the protein.

## Data Availability

Not applicable.

## Abbreviations

3-MA	3-methyladenine
A2780	Human cells from ovarian carcinoma
A549	Human cells derived from non-small cell lung carcinoma
Akt	Protein kinase B
AMPK	Adenosine monophosphate-activated protein kinase
ATG	Autophagy-related protein
*ATG5*	Autophagy-related gene 5
*ATG7*	Autophagy-related gene 7
ATP	Adenosine triphosphate
Bcl2	B-cell lymphoma 2
BGC-823	Human cells derived from gastric carcinoma
BiP	Binding immunoglobulin protein
BJ	Human primary fibroblasts from foreskin
BRAF	V-raf murine sarcoma viral oncogene homolog B1
Caco-2	Human cells from colorectal carcinoma
CGs	Cardiac glycosides
CHOP	CCAAT/enhancer-binding protein homologous protein
COVID-19	Coronavirus disease 2019
CT26	*N*-nitroso-*N*-methylurethane-induced, undifferentiated colon carcinoma cell line
DAMPs	Damage(Danger)-associated molecular patterns
EGFR	The epidermal growth factor receptor
eIF2α	Eukaryotic translation initiation factor 2α
ER	Endoplasmic reticulum
ERK1/2	Extracellular signal-regulated protein kinases 1 and 2
GES-1	Gastric mucous epithelium cells
Grb2	Growth factor receptor-bound protein 2
GRP78	Binding immunoglobulin protein
H460	Human cells derived from non-small cell lung carcinoma
HA22T	Human cells from hepatocellular carcinoma
HCCLM3	Human cells from hepatocellular carcinoma
HCMV	Human cytomegalovirus
HCT 116	Human cells from colorectal carcinoma
HeLa	Human cells derived from cervical carcinoma
Hep3B	Human cells from hepatocellular carcinoma
HepG2	Human cells from hepatocellular carcinoma
HT-29	Human cells from colorectal carcinoma
Huh7	Human cells from hepatocellular carcinoma
IC_50_	Half-maximal inhibitory concentrations
ICD	Immunogenic cell death
I/R	Ischemia/reperfusion
IGROV-1	Human cells from ovarian carcinoma
IP_3_	Inositol 1,4,5-trisphosphate
JNK	c-Jun N-terminal kinases
LC3-II	Microtubule-associated protein 1A/1B-light chain 3-II
LN-229	Human cells derived from glioblastoma
MCF-7	Human cells from breast carcinoma
Mcl-1	Myeloid-cell leukemia 1
MDA-MB-231	Human cells from breast carcinoma
MEK	Mitogen-activated protein kinase
MEK 1/2	Mitogen-activated protein kinase kinases 1 and 2
MAPK	Mitogen-activated protein kinase
MHCC97H	Human cells derived from hepatocarcinoma
mTOR	Mammalian target of rapamycin
NHE	Na^+^/H^+^-exchanger
NF- κB	Nuclear factor kappa-light-chain-enhancer of activated B cells
NKA	Sodium-potassium ATPase
NMTC	Non-medullary thyroid cancer
OCCC	Ovarian clear cell carcinoma
PA	Palmitic acid
PANC-1	Human cells from pancreatic carcinoma
PARP	Poly(adenosine diphosphate-ribose) polymerase
PAS	Phagophore assembly site
PERK	Protein kinase R-like endoplasmic reticulum kinase
PI3K	Phosphoinositide 3-kinase
PLC	Phospholipase C
PP2	1-*tert*-butyl-3-(4-chlorophenyl)-1H-pyrazolo[3,4-d]pyrimidin-4-amine)
Rab7	Ras-related protein
Raf	Rapidly accelerated fibrosarcoma protein
Ras	Rat sarcoma virus protein
RAW 264.7	Mouse macrophages
ROS	Reactive oxygen species
SCC2095	Cells derived from oral squamous carcinoma
SGC 7901	Human cells derived from gastric carcinoma
SH-SY5Y	Human cells from neuroblastoma
Shc	Src homology 2 domain-containing transforming protein
SK-OV-3	Human cells from ovarian carcinoma
siRNA	Small interfering ribonucleic acid (siRNA
Sos	Son of sevenless protein
Src	Proto-oncogene tyrosine-protein kinase Src
Tau	Tubulin-associated unit
TFEB	Transcription factor EB
TOV-21G	Human cells from ovarian carcinoma
TRAIL	Tumor necrosis factor-related apoptosis-inducing ligand
U-2 OS	Human cells from osteosarcoma
U-87 MG	Human cells derived from glioblastoma
ULK1	Unc-51 like autophagy activating kinase
Wnt	Wingless-related integration site protein
